# Transumbilical single-site laparoscopic treatment of small intestinal cavernous hemangioma in child: a case report

**DOI:** 10.3389/fonc.2024.1360557

**Published:** 2024-02-29

**Authors:** Meng Kong, Weiqiang Liu, Yuexia Bai, Jinhua Jia, Chuanyang Liu, Shisong Zhang

**Affiliations:** ^1^ Department of Pediatric Surgery, Children’s Hospital Affiliated to Shandong University, Jinan, China; ^2^ Department of Pediatric Surgery, Jinan Children’s Hospital, Jinan, China; ^3^ Department of Pediatric Surgery, Zhucheng Women and Children’s Hospital, Weifang, China; ^4^ Department of Pathology, Children’s Hospital Affiliated to Shandong University, Jinan, China

**Keywords:** cavernous hemangioma, children, small intestine, laparoscopy, surgery

## Abstract

**Background:**

While hemangiomas are the most commonly occurring benign vascular tumors, their occurrence in the gastrointestinal system is rare. This case report presents a unique instance of small intestinal hemangioma in a pediatric patient.

**Case description:**

A 21-month-old girl was admitted to the hospital with a history of “recurrent blood in the stool for one year and anemia for five months.” Upon evaluation at our facility, abdominal color ultrasound and enhanced CT scans revealed a protruding mass in the wall of the small intestine, leading to a preliminary diagnosis of small intestinal hemangioma. Subsequent single-site umbilical laparoscopic exploration identified a tumor measuring approximately 6cm×2.5cm×1.2cm on the jejunum wall. Consequently, segmental resection of the intestine was performed, and the postoperative pathological diagnosis confirmed cavernous hemangioma.

**Conclusion:**

Small intestinal hemangiomas, particularly in pediatric patients, are exceptionally rare and challenging to diagnose as the cause of gastrointestinal bleeding prior to surgery. Hence, small intestinal hemangiomas should be considered in such cases. Laparoscopic surgical resection emerges as the optimal approach for addressing small intestinal hemangiomas.

## 1 Introduction

Hemangioma is a common benign vascular tumor that originates from embryonic mesoderm tissue. It is characterized by the rapid proliferation of endothelial cells, which can vary in form, location, and size (1, 2). Hemangiomas commonly occur in the skin, mucosa, and liver, while gastrointestinal hemangiomas are rare, accounting for only 0.05% of all hemangiomas (3). Small intestinal hemangioma is a rare type of gastrointestinal hemangioma, representing 7%-10% of all benign tumors in the small intestine (3, 4). These hemangiomas can present as single or multiple lesions, with the most frequent clinical manifestations including gastrointestinal bleeding, anemia, abdominal pain, and other symptoms. In some cases, complications such as intussusception, intestinal obstruction, and intestinal perforation may arise (5).Here, we present a case report of a small intestinal cavernous hemangioma in a 21-month-old female child who primarily exhibited symptoms of hematochezia and anemia. We successfully performed laparoscopic surgery to remove the affected bowel segment. The aim of this report is to enhance the understanding of small intestinal hemangiomas in children, thereby reducing misdiagnosis and missed diagnoses. This case report adheres to the SCARE criteria (6) and has obtained informed written consent from the patient’s family.

## 2 Case report

### 2.1 General information

A 21-month-old female patient was admitted to the hospital due to “recurrent blood in the stool for 1 year and anemia for 5 months.” There was no apparent trigger for the onset of bloody stools a year ago. Initially, the stools were tarry and persisted for about 2 days, occurring approximately 2 times a day, before transitioning to yellow sticky stools. Subsequently, the child experienced this approximately 2-3 times a month without any associated discomfort and did not receive any specific treatment.

Five months prior to admission, the patient was taken to a local hospital again due to “blood in the stool.” At that time, her hemoglobin level was measured at 63g/L, and stool occult blood test was positive. Following oral iron treatment for 3 months, the hemoglobin level increased to 119g/L, after which the medication was discontinued. Approximately half a month before admission to our hospital, the patient sought medical attention at a local hospital due to “fever,” during which her hemoglobin was found to be 49g/L. She received hemostatic treatment, red blood cell transfusion, and other interventions to correct the anemia. After 5 days of treatment, her hemoglobin level rose to 95g/L. The current admission to our hospital was prompted by continued blood in the stool, following an outpatient clinic visit. Previous History: There were no significant findings in the personal or family history. In addition, the child was born at term with no history of perinatal hypoxia.

### 2.2 Auxiliary examination

**Figure 1:**
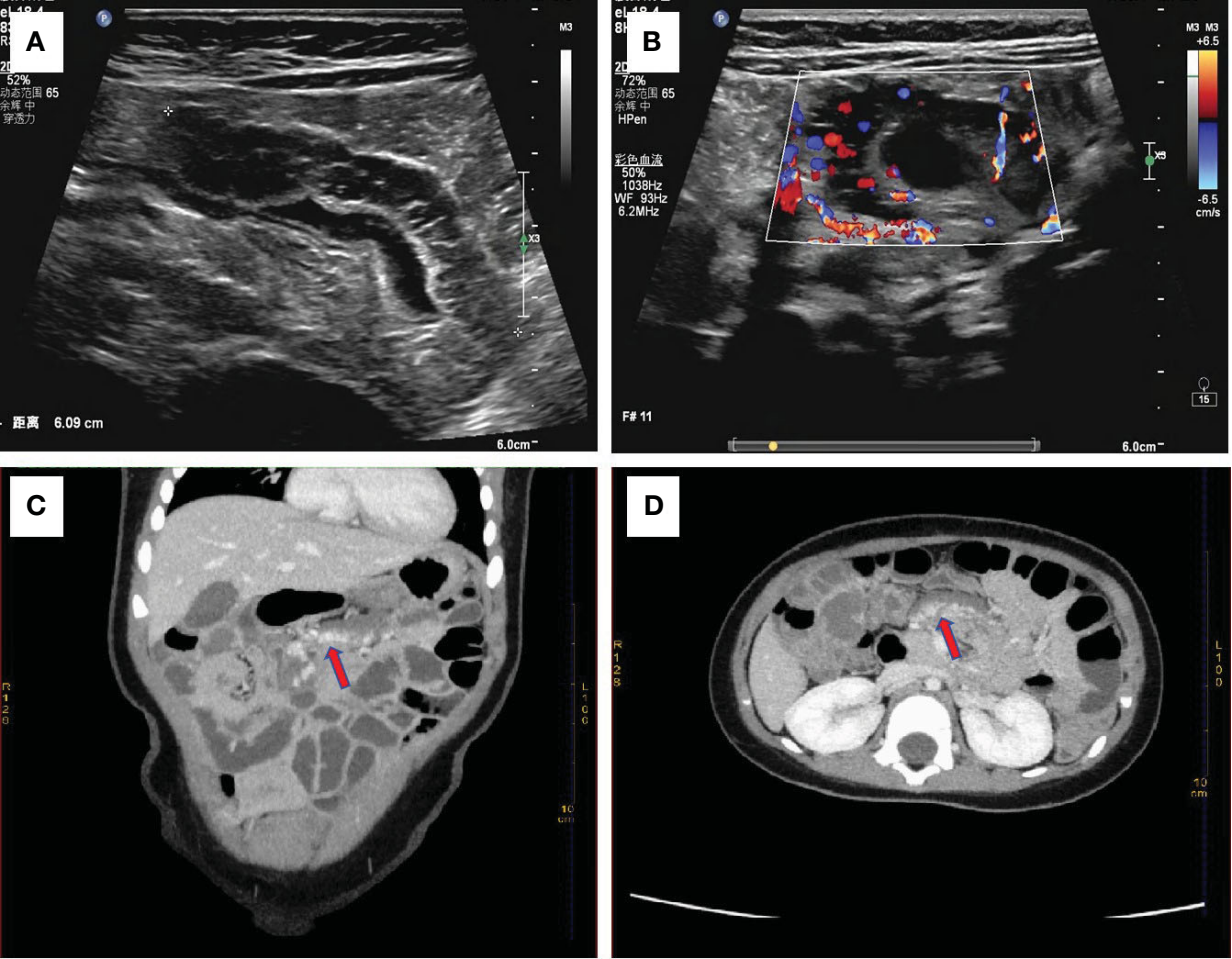
**(A)** Abdominal color ultrasonography shows stiff morphology of small intestine with slightly obstructed passage of contents, suggesting segmental venous malformation. **(B)** Abundant blood flow signals are seen around the wall of the small intestine. Enhanced abdominal CT shows focal thickening of the small intestine wall with tortuous, chaotic and high-density vascular shadows. The blood supply artery is from the superior mesenteric artery. This indicated venous malformation of the small intestine **(C)** with coronary vascular signs and **(D)** with cross-sectional vascular signs.

Physical Examination: The patient had a flat abdomen with no signs of gastrointestinal or abdominal wall varicose veins. There was no tenderness, rebound pain, or muscle tension across the entire abdomen, and no obvious masses, liver, spleen, or subcostal lesions. Additionally, there were no skin rashes or hemorrhagic spots on the body. Laboratory Examination: Blood cell analysis revealed a red blood cell count of 3.68×10^12^/L, hemoglobin content of 87.00g/L, and reticulocyte count of 0.15×10^12^/L. Blood biochemistry showed a total protein level of 56.3g/L, globulin level of 16.3g/L, white bulb ratio of 2.5, total bilirubin level of 5.2μmol/L, direct bilirubin level of 2.6μmol/L, and cholinesterase level of 7603U/L. Serum tumor markers were not tested. Imaging Examination: Abdominal color ultrasonography revealed segmental asymmetrical tube wall thickening in the left upper small intestine of the umbilical part in the abdomen. The layers disappeared, and there was a honeycomb echoless area inside measuring approximately 6.0cm in length and 1.2cm in thickness, with patchy blood flow signal shown inside. This section of intestinal tube was stiff, and the passage of contents was slightly blocked. However, the rest of the intestine peristalsis was normal without signs of dilation or fluid accumulation. There was no significant thickening of the mesentery, and no echo of an abnormal mass was detected. These findings were suggestive of a left upper jejunal segmental venous malformation (cavernous hemangioma) of the umbilical cord (**Figures 1A, B**). Abdominal CT plain scan and enhanced scan showed that the local lumen of the left upper abdomen jejunum was stiff, and the tube wall was thickened. After enhanced scan, tortuous and chaotic blood vessels were seen in the intestinal wall, with the blood supply artery coming from the superior mesenteric artery. The preliminary diagnosis was a local intestinal wall thickening in the left jejunum of the upper abdomen with multiple tortuous vascular shadows, suggestive of an intestinal venous malformation (**Figures 1C, D**). After detailed preoperative physical examination and auxiliary examination (abdominal color ultrasound or CT), we found no hemangioma in other parts of the child’s body.

### 2.3 Surgical procedure

**Figure 2:**
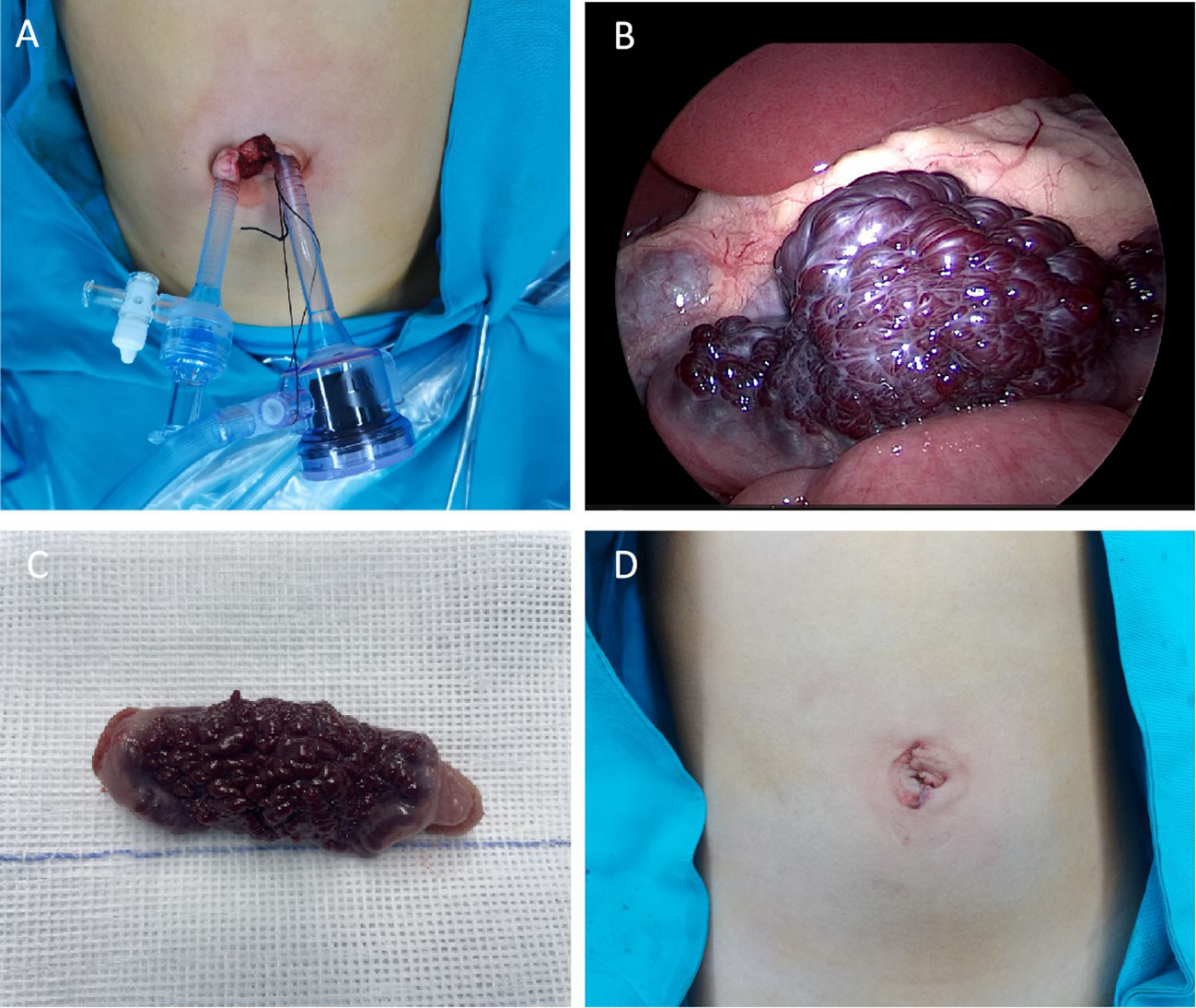
**(A)** Location of Trocar in the umbilical cord **(B)** Microscopic view of small intestinal hemangioma **(C)** Gross view of the resected small intestinal hemangioma marked tortuous dark red vessels with obvious congestion in the serous membrane. **(D)** postoperative appearance of the umbilical cord.

Based on the medical history, physical examination, and auxiliary examination, we considered the possibility of small intestinal hemangioma. Due to the patient’s young age and the size of the tumor, we decided to perform a minimally invasive transumbilical laparoscopic surgery. The surgical procedure was carried out as follows: 1)The patient was properly anesthetized and positioned horizontally. The operative area was disinfected, and a sterile towel was placed. 2)A 2.0cm longitudinal incision was made on the skin of the umbilical cord. The subcutaneous tissue was separated on both sides of the incision until reaching the muscular layer. 3)A 5mm Trocar was inserted on each side to establish CO2 pneumoperitoneum, with a pressure of 6mmHg and a flow rate of 2L/min. 4)We put the laparoscope on the child’s head and the surgeon stands on the right side of the child. Then change the child from a horizontal position to a head low and feet high position. Laparoscopic light source was placed in the right Trocar for observation, and laparoscopic intestinal forceps were placed in the left Trocar (**Figure 2A**). We used laparoscopic intestinal forceps to push up the colon, exposing the duodenal Treitz’s ligament. After locating the Treitz’s ligament of the duodenum, the probe was conducted from the proximal end of the duodenum to the distal end. In particular, laparoscopic procedures are performed by the surgeon alone. In particular, laparoscopic procedures are performed by the surgeon alone.5)The small intestine was explored from the duodenal Treitz’s ligament to the distal end. An abnormal dark red vessel, measuring approximately 6cm×2.5cm×1.2cm, was found on the wall of the small intestine, 20cm away from the Treitz’s ligament (**Figure 2B**). The lesion was mainly located at the mesangial margin. No abnormalities were observed in the rest of the small intestine during further exploration. 6)The diseased segment of the small intestine was grasped and fixed with forceps. The umbilical Trocar and endoscope were then withdrawn. 7)The umbilical incision was enlarged to access the deep tissue. The diseased segment of the small intestine and the adjacent normal segment were removed. The mesenteric vessels were ligated and sutured (**Figure 2C**). 8)The diseased segment of the small intestine was completely removed, and an end-to-end intestinal anastomosis was performed using discontinuous sutures with a 5-0 absorbable suture. 9)The anastomosis was carefully checked for patency and absence of leakage. The mesenteric hematoma was repaired. 10)The small intestine was returned to the abdomen. No bleeding was observed, and the umbilical incision was closed layer by layer (**Figure 2D**). The operation was successfully completed.

### 2.4 Pathological findings

**Figure 3:**
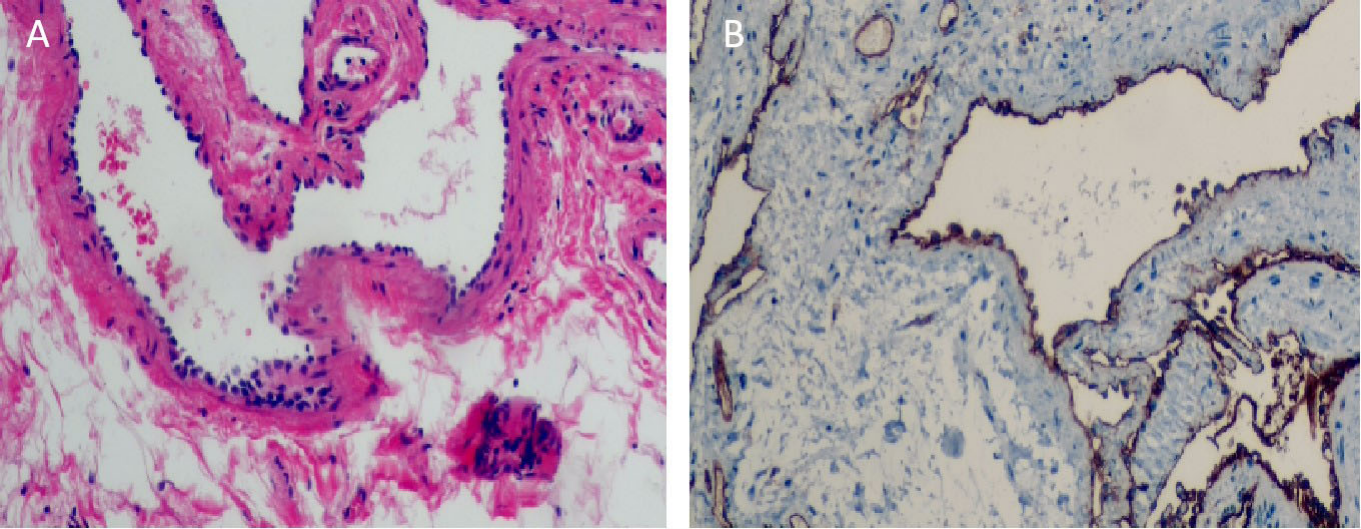
**(A)** Under electron microscope, the submucosa showed irregular dilated vessels with multiple lumens. The vascular wall was lined with small and deeply stained endothelial cells, and the vascular endothelial hyperplasia was obvious (Hematoxylin and eosin staining,×100). **(B)** Immunohistochemical staining results showed that cells arranged in the vascular space were CD31 positive. (Immunohistochemical staining, ×100).

**Figure 4:**
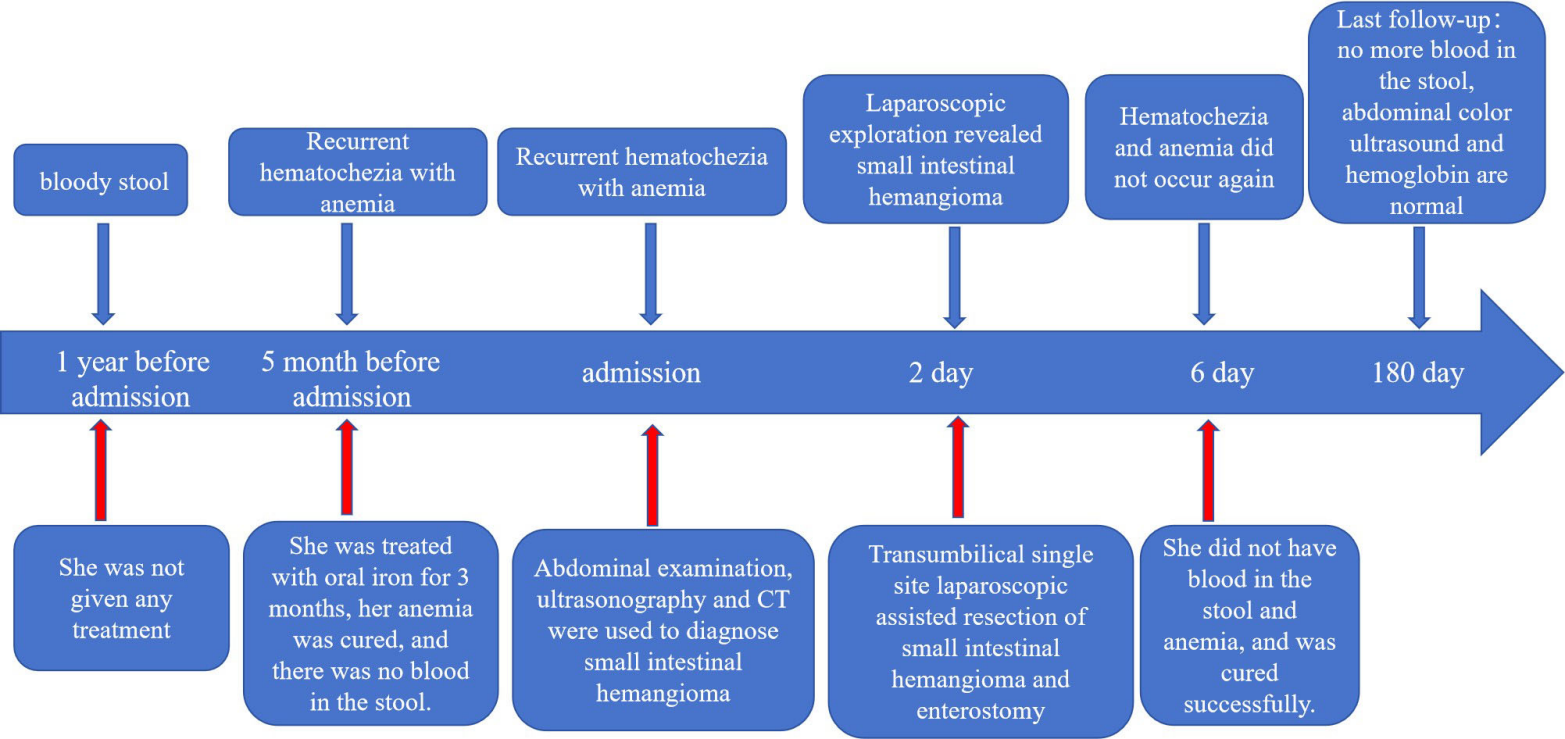
Complete timeline, including diagnosis (surgery) and treatment.

Upon visual inspection, a section of the intestinal duct measuring 8.0cm in length and 2cm-2.5cm in diameter was observed. On the serosal surface, there was a slightly raised purplish red area measuring 4.5cm×2cm. The incisal margin, located 1cm away, revealed a granular intestinal mucosa measuring 4.5cm×1.5cm. The purplish red area appeared slightly spongy and soft in texture. Histopathological analysis revealed that the tumor was a cavernous hemangioma that had invaded the entire intestinal wall. Ganglion cells were detected in both the muscle and submucosa layers. No hemangioma components were identified at the incisal margins on both sides of the intestinal duct (**Figure 3A**). Immunohistochemical staining showed positive expression of the vascular endothelial biomarker CD31 (**Figure 3B**). Three months after the operation, there were no reports of hematochezia (bloody stools), and abdominal color ultrasonography did not reveal any abnormalities. Blood cell analysis indicated a red blood cell count of 4.77×10^12^/L and a hemoglobin level of 129g/L (**Figure 4**).

## 3 Discussion

Hemangioma of the small intestine is a rare congenital hemangiomatoma of the gastrointestinal tract characterized by vascular dysplasia originating in the submucosal vascular plexus and extending into the muscular or serosal layers. It can be single or multiple, occurring at any age and in any segment of the small intestine, with the jejunum being the most common site (7).The typical clinical manifestations include acute and chronic gastrointestinal bleeding, leading to unexplained anemia, as well as rare symptoms such as abdominal pain, intestinal obstruction, intussusception, and intestinal perforation (8). In a literature review of 25 cases of small intestinal hemangioma, Fu et al. (4) found that 44% were primarily treated for black stool, followed by 28% presenting with anemia. The children in this case came to our hospital due to hematochezia and anemia. Notably, the youngest age reported in previous literature was 6 years old, with the majority of cases occurring in adults (3), whereas the children in this case exhibited symptoms of hematochezia and anemia at a relatively early age, at only 21 months old.

Small intestinal hemangiomas are classified into three types based on histopathology and vessel diameter: cavernous vascular type (comprising one or more layers of endothelial cells lining blood-filled sinuses, infiltrating long segments of the intestinal duct and mesentery); capillary type (thin-walled capillaries composed of vascular endothelial cells, filled with blood); and mixed vascular type. Some cases may exhibit focal calcification, thrombus, and hyaloid lesions, indicating degenerative changes. Among these, cavernous hemangioma is the most common, infiltrating the large intestine and mesentery (9). Tumor size can range from a few millimeters to a few centimeters. In this case, both intraoperative and postoperative pathology confirmed that the tumor was cavernous and diffusely spread across the intestinal wall, composed of numerous blood sinuses filled with blood that were visible in the serosal layer.

Small intestinal hemangioma, like other benign gastrointestinal tumors discovered incidentally, is often misdiagnosed and missed during early diagnosis due to the absence of specific symptoms. The correct diagnosis rate during surgery is only 25.6%. Detecting this disease before surgery is difficult, and surgeons need to make a definitive diagnosis during or after exploratory laparotomy (10). When differentiating small intestinal hemorrhage in children, it is important to distinguish between small intestinal diverticulum, intestinal duplication malformations, inflammatory bowel disease (ulcerative colitis, Crohn’s disease), eosinophilic gastroenteritis, duodenal ulcer, and intestinal polyps. Various examination methods are used to detect small intestinal bleeding, including abdominal CT/MRI, small intestinal angiography, gastroenteroscopy, colonoscopy, capsule endoscopy, and double balloon colonoscopy (11). In recent years, abdominal color ultrasound has shown improvement in diagnostic capabilities. Initial screening with abdominal color ultrasound is recommended due to its convenience and non-invasive nature. CT imaging of small intestinal hemangiomas typically shows a distinct mass in the mucosal tissue with clear boundaries. Nodular enhancement is observed in the arterial stage, patellar enhancement in the venous stage, and partial filling of lesions in the delayed stage. Obvious dilated vascular images may be seen in the peripheral mesentery if complications such as intussusception, intestinal obstruction, or intestinal perforation are present (12). Abdominal CT can also reveal related gastrointestinal angiomatosis, such as Klippel-Trenaunay syndrome, blue rubber bleb nevus syndrome (BRBNS), and Maffucci syndrome. These conditions are characterized by a diffuse distribution in the intestinal wall and mesentery (13). Aside from abdominal color ultrasonography and CT imaging, capsule endoscopy or double balloon colonoscopy may be considered for diagnosis if available in the hospital (14, 15). Capsule endoscopy is a non-invasive method with a high diagnostic rate for overt and persistent bleeding, but its effectiveness is limited during acute bleeding. It has some limitations, such as inaccurate positioning, inability to perform tissue biopsy, and capsule retention (16). On the other hand, double balloon colonoscopy is an invasive and highly sensitive diagnostic tool that allows for therapeutic and diagnostic interventions. Its use has been shown to significantly improve the diagnosis rate of the disease (17). However, these procedures are not widely available. In cases where capsule endoscopy or double balloon enteroscopy is not feasible, laparoscopic exploration may be the most suitable choice, even if no lesions are found. Laparoscopic exploration allows for simultaneous diagnosis and surgical resection (18).

The continuous advancements in science and technology have progressively steered surgical practices toward minimally invasive and precision-oriented approaches. Laparoscopic technology epitomizes this shift toward minimally invasive surgery, gaining widespread acceptance among both medical professionals and patients for its reduced invasiveness. Particularly in pediatric surgery, there’s a common goal shared by surgeons and patients alike to minimize scarring. The navel, being a “natural channel,” has garnered attention as an ideal site for minimizing surgical scars (19). In our case, we utilized laparoscopic exploration to first identify the lesion site within the small intestine, followed by the ex vivo removal of a small intestinal hemangioma through an incision at the umbilical site. This approach amalgamates the benefits of traditional surgery with those of laparoscopic techniques, offering a single-incision method that serves both diagnostic and therapeutic purposes. This strategy circumvents the uncertainties associated with open exploratory laparotomies. Our adoption of transumbilical single-site dual-channel laparoscopic-assisted resection for small intestinal hemangiomas enhances precision. This method not only facilitates accurate diagnosis but also significantly reduces the exposure of the intestinal tube and the duration of exploration. Consequently, it notably diminishes the likelihood of postoperative intestinal adhesion, shortens the operation time, and minimizes the trauma inflicted on children by surgical and anesthetic procedures. This approach results in less trauma, minimal blood loss, fewer complications, and a rapid recovery. Importantly, it ensures a minimal postoperative scar appearance, achieving an excellent aesthetic outcome (20). Furthermore, by performing the cavernous hemangioma resection and intestinal anastomosis outside the abdominal cavity, the surgical procedure becomes more intuitive. This allows for the complete removal of the hemangioma, ensuring that surrounding pathological tissues are not overlooked, thereby preventing postoperative recurrence and re-bleeding.

In our case, we have distilled several key insights: The abdominal cavity in children is compact, and the positioning of the intestinal tubes can vary, making it challenging to locate lesions via endoscopy. Preoperative abdominal ultrasound and CT scans suggested that the lesions were near the proximal end of the duodenum. To address this, we positioned the child with their head elevated and legs lowered, using intestinal forceps to lift the colon and fully expose the duodenum. This approach allowed us to methodically inspect the intestine from the proximal to the distal end, ensuring no lesions were overlooked. Besides, the placement of two Trocars through the umbilicus can lead to interference between them, complicating the operation due to conflicts between the light source and the operating forceps within the abdominal cavity. We recommend liberating the subcutaneous tissue around the incision as much as possible to maximize the distance between the two Trocars. Additionally, adjusting the Trocars to different heights-one deeper, one shallower-can further minimize the risk of collision. Proficient laparoscopic skills and a stable surgical team are crucial for success. Ideally, the laparoscopic procedure should be conducted by a single surgeon to enhance coordination between the visual field and the manipulation of instruments, thereby reducing the likelihood of interference. In summary, performing laparoscopic resection of small intestinal hemangiomas through the “natural orifice” of the umbilicus not only leverages the benefits of minimal invasiveness, effective outcomes, rapid postoperative recovery, and discreet concealment of the surgical site but also fulfills the aesthetic preferences of children and their parents for an abdomen free of visible scars. This approach is particularly advantageous for pediatric patients experiencing unexplained intestinal bleeding, offering superior diagnostic and therapeutic results.

Surgical resection of the affected bowel is currently the preferred treatment for intestinal hemangioma (21). Since hemangiomas are benign tumors that do not spread to lymph nodes or distant tissues and organs, local resection is sufficient. In some cases where polypoid lesions are identified by endoscopy, endoscopic resection or cauterization may be performed. However, these treatments carry risks of uncontrolled gastrointestinal bleeding, intestinal perforation, and a high recurrence rate (22). Drug therapy, such as oral propranolol, has been reported as a successful treatment for neonatal gastric hemangioma (23). However, surgical resection is still the preferred treatment for symptomatic hemangiomas that present with life-threatening bleeding, intussusception, intestinal obstruction, and intestinal perforation. Small intestinal hemangiomas generally have a good prognosis, and recurrent cases are rare (24). As in our case, we decided to successfully cure the patient by laparoscopy and resection of the diseased intestinal tube.

## 4 Conclusions

In conclusion, small intestinal hemangiomas are a rare but significant cause of gastrointestinal bleeding, often missed due to their non-specific symptoms. Preoperative diagnosis can be achieved through abdominal color ultrasound and abdominal enhanced CT, and laparoscopic surgery is an effective treatment option. Endoscopic treatment should be approached with caution and may be suitable for multiple and relatively small lesions, while laparoscopic resection is preferred for large and diffuse lesions.

## Patient’s perspective

Patient’s parents: “Thank you very much for the skill of the doctor. The doctor successfully cured our child’s bloody stool and anemia with minimally invasive surgery. I can’t believe our baby has a hemangioma on his bowel”.

## Data availability statement

Publicly available datasets were analyzed in this study. This data can be found here: Corresponding Author.

## Ethics statement

The studies involving humans were approved by Ethics Committee of Children’s Hospital Affiliated to Shandong University. The studies were conducted in accordance with the local legislation and institutional requirements. Written informed consent for participation in this study was provided by the participants’ legal guardians/next of kin. Written informed consent was obtained from the individual(s), and minor(s)’ legal guardian/next of kin, for the publication of any potentially identifiable images or data included in this article.

## Author contributions

MK: Writing – original draft, Writing – review & editing. WL: Writing – review & editing. YB: Writing – review & editing. JJ: Writing – review & editing. CL: Writing – review & editing. SZ: Writing – review & editing.
